# Minimization of Entropy Generation Rate in Hydrogen Iodide Decomposition Reactor Heated by High-Temperature Helium

**DOI:** 10.3390/e23010082

**Published:** 2021-01-08

**Authors:** Rui Kong, Lingen Chen, Shaojun Xia, Penglei Li, Yanlin Ge

**Affiliations:** 1 College of Power Engineering, Naval University of Engineering, Wuhan 430033, China; kongrui3465@163.com (R.K.); 15994280441@139.com (S.X.); lipenglei19940401@hotmail.com (P.L.); 2 Institute of Thermal Science and Power Engineering, Wuhan Institute of Technology, Wuhan 430205, China; geyali9@hotmail.com; 3 School of Mechanical & Electrical Engineering, Wuhan Institute of Technology, Wuhan 430205, China

**Keywords:** hydrogen iodide decomposition, tubular reactor, entropy generation rate minimization, finite-time thermodynamics, optimal control theory

## Abstract

The thermochemical sulfur-iodine cycle is a potential method for hydrogen production, and the hydrogen iodide (HI) decomposition is the key step to determine the efficiency of hydrogen production in the cycle. To further reduce the irreversibility of various transmission processes in the HI decomposition reaction, a one-dimensional plug flow model of HI decomposition tubular reactor is established, and performance optimization with entropy generate rate minimization (EGRM) in the decomposition reaction system as an optimization goal based on finite-time thermodynamics is carried out. The reference reactor is heated counter-currently by high-temperature helium gas, the optimal reactor and the modified reactor are designed based on the reference reactor design parameters. With the EGRM as the optimization goal, the optimal control method is used to solve the optimal configuration of the reactor under the condition that both the reactant inlet state and hydrogen production rate are fixed, and the optimal value of total EGR in the reactor is reduced by 13.3% compared with the reference value. The reference reactor is improved on the basis of the total EGR in the optimal reactor, two modified reactors with increased length are designed under the condition of changing the helium inlet state. The total EGR of the two modified reactors are the same as that of the optimal reactor, which are realized by decreasing the helium inlet temperature and helium inlet flow rate, respectively. The results show that the EGR of heat transfer accounts for a large proportion, and the decrease of total EGR is mainly caused by reducing heat transfer irreversibility. The local total EGR of the optimal reactor distribution is more uniform, which approximately confirms the principle of equipartition of entropy production. The EGR distributions of the modified reactors are similar to that of the reference reactor, but the reactor length increases significantly, bringing a relatively large pressure drop. The research results have certain guiding significance to the optimum design of HI decomposition reactors.

## 1. Introduction

Hydrogen energy is a kind of renewable and clean energy, and it is necessary to research and develop hydrogen energy under the background of fossil energy shortage and greenhouse effect. The thermochemical sulfur-iodine (S-I) cycle can couple with a nuclear reaction and solar heating [1,2,3]; then the overall thermal efficiency can reach more than 50% [4], so it has great development potential. The thermochemical S-I cycle for hydrogen production was first proposed by the General Atomics Company in the 1970s, and its reliability was analyzed and demonstrated [5,6]. In the following decades, many related researches have been successfully implemented, and the process method of the S-I cycle were further developed and improved [7,8,9].

**Figure 1:**
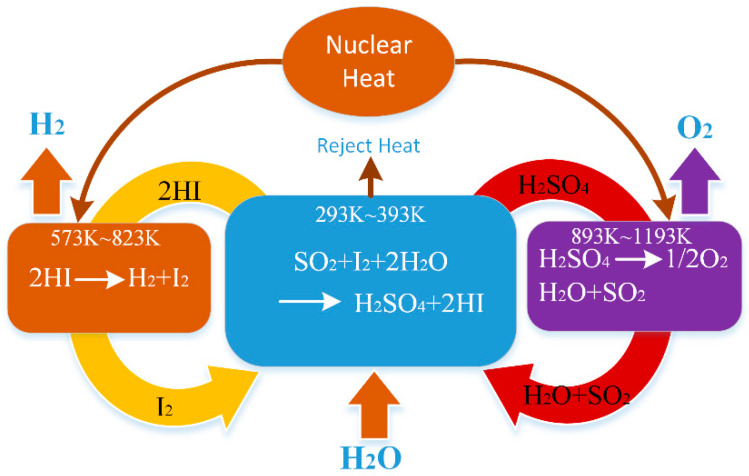
Schematic diagram of the S-I cycle.

There are three main chemical reactions in the thermochemical S-I cycle, which are the Bunsen reaction, sulfuric acid decomposition and HI decomposition reactions. Figure 1 shows the schematic diagram of the S-I cycle. For the HI decomposition process, comprehensive researches have been implemented including catalyst performance evaluation [10,11], reaction kinetics analysis [12,13,14,15] and reactor numerical simulation [16,17,18]. The conversion rate of HI decomposition reaction is low, which restricts the hydrogen yield and thermal efficiency of the S-I cycle system, and the choice of decomposition temperature must take into account the heat resistance of the material and the thermal efficiency of the device. The HI decomposition process is considered to be one of the most critical steps in hydrogen production process using S-I cycle. A large amount of H_2_O exists in the process of distilling HI gas from the HIx solution, the H_2_O and HI need to be heated during the reaction and the unreacted HI gas after the decomposition reaction needs to be circulated and reacted again, so this process requires a lot of heat [4]. Therefore, it is necessary to further study and analyze the thermodynamic irreversibility in the HI decomposition process.

Finite-time thermodynamics (FTT) has made significant progress in physics and engineering fields since the mid-1970 s [19,20,21,22,23,24,25,26,27,28,29,30,31,32,33], and the aim is to reduce the irreversibility of systems under finite time and size constraints. The applications of FTT include the researches of optimal performances [34,35,36,37,38,39,40,41,42,43,44,45,46,47,48,49,50] and optimal configurations [51,52,53,54,55,56,57,58,59,60,61,62,63,64,65,66,67,68] for various thermodynamic processes, devices and cycles.

FTT theory was also applied in chemical reaction processes; see the review article [69]. Some scholars [70,71,72,73] have conducted a lot of researches by taking the maximum production rate (PR) and minimum entropy generation rate (EGR) as optimization targets for chemical reaction processes. Måsson and Andresen [70] first applied FTT theory to optimize synthetic ammonia reaction, and the temperature distribution curve of the reaction mixture was solved with ammonia production rate maximization (PRM) as the optimization goal under the given inlet status. Chen et al. [71] established the thermodynamic model for removing CO_2_ from acidified seawater with hollow fiber membrane contacts, and obtained the optimal configuration of CO_2_ concentration with the EGRM in the mass transfer process as the optimization objective. Chen et al. [72] also investigated the reaction of carbon dioxide and hydrogen to synthesize olefins, and analyzed the effect of reactor structural parameters on specific EGRs; the specific EGR could be reduced by 10.04% and 24.78% under the optimal catalyst bed density and pipe diameter, respectively. Li et al. [73] studied the steam methane reforming reactor with hydrogen PRM as the optimization goal, and obtained the optimal wall temperature distribution and inlet pressure. The hydrogen PR of the optimal reactor increase by 11.8% was compared with the reference value. Li et al. [74] further established the model of the steam methane reforming reactor heated by molten salt, and the total EGR could be reduced by 22% compared with the reference value, by optimizing the mixture and molten salt inlet parameters. Nummedal et al. [75] investigated the steam reforming reaction of methane with EGR minimization (EGRM) as the objective function under the constraint of fixed hydrogen PR, and optimized the gas inlet temperature, the external heat source and the inlet mixture composition by the nonlinear programming method. Wang et al. [76] optimized the decomposition process of sulfuric acid by the non-linear program design method with the goal of SO_2_ PRM, and the PR was improved by 7% under optimized temperature and pressure profiles. The method of nonlinear programming is to discretize continuous variables, so the results are usually approximate solutions. The optimal control method is relatively complex, but the results are relatively accurate. Johannessen and Kjelstrup [77] first used this method to solve the optimization problem of the sulfur dioxide oxidation reaction, and obtained the optimal heat source temperature distribution curves at different tube lengths with the total EGRM as the optimization goal. The authors Vander Ham et al. [78] studied the decomposition of sulfuric acid in a tubular reactor, obtained the optimal helium temperature distribution under the condition of EGRM, the total EGR is 26% lower than the that of the reference reactor heated by helium counterflow. Zhang et al. [79] investigated olefin synthesis reactors with the optimal control method; the optimal heat reservoir temperature profiles were obtained with EGRM as the optimization target under fixed or free inlet temperature and CO_2_/H_2_ ratio. Li et al. [80] analyzed the synthesis of methanol by CO_2_ and hydrogen under the constraint of fixed methanol PR, and the optimal reactor heat source temperature distribution was obtained with the goal of EGRM; the EGR value in optimal reactor was reduced by 20% compared with the reference reactor using a constant heat source. Kong et al. [81] studied the HI decomposition reactor with a fixed hydrogen PR as the constraint, and the optimal heat source temperature distribution curves were obtained using the optimal control method under the conditions of fixed and free tube length; the total EGR was reduced by 51.3% and 57.6% compared with that in the reference reactor heated with linear heat source temperature, respectively. Zhang et al. [82] used the optimal control method to investigate reverse water gas shift reactors with EGRM as the optimization target, and obtained the optimal configuration of the reactor under different boundary conditions and lengths. On the basis of previous research work, researches involving multiple optimization objectives and multiple reaction processes have been reported. Zhang et al. [83] established the model of reverse water gas reactor heated by high temperature helium, and carried out multi-objective optimization with minimum radial temperature difference and maximum conversion rate. Cao et al. [84] studied the multi-objective optimization of EGR in mass transfer and chemical reaction processes. Sun et al. [85] investigated the sulfuric acid decomposition reaction and carried out multi-objective optimization with minimum EGR and maximum SO_2_ PR. Zhang et al. [86] established a reaction distillation model of Fetol synthesis and studied the multi-objective optimization problem in the reaction process. Avellaneda et al. [87] carried out multi-objective optimization on the heat and mass transfer process of convective flows in the flow passage. Magnanellia et al. [88] investigated the membrane device to separate carbon dioxide from natural gas, and the research results showed that EGR due to mass transfer in the membrane permeable process could be minimized by controlling the partial pressure and total pressure of the gas components on the permeable side. Kingston et al. [89] studied the air separation process of the packed distillation column and obtained the optimal temperature distribution curve of heat transfer between the column and its surroundings with the aim of EGRM. Korpyś et al. [90] applied an entropy optimization criterion to the methane catalytic combustion process and analyzed the influence of different catalysts on the EGR in the reactor. Kizilova et al. [91] optimized the channel shape of the fuel cell with the minimum EGR caused by viscous flow. Yang et al. [92] studied the heat transfer process and EGR of the two-layer porous media tube, and analyzed the local EGR under different parameters. Li et al. [93] simulated the decomposition process of methane hydrate and analyzed the heat transfer characteristics and entropy generation under different pressure conditions. Under the constraint of fixed hydrogen PR, the EGRM was taken as the objective to optimize the HI decomposition reaction process, and the ultimate goal was to reduce the irreversibility of the reaction process and reduce the energy quality loss.

There are three methods to reduce entropy generation in a chemical reactor: optimizing the distribution of EGR, increasing the reactor length and improving the catalyst performance [78]. The first method is to optimize the heat source temperature distribution and adjust the distribution of local EGR; the second method is to increase the heated area and reduce the average heat flux in the heat transfer process; and the third method is to increase the reaction transmission coefficient and increase the chemical reaction rate.

In this article, the HI decomposition reaction process will be further optimized using FTT theory, in order to explore the potential of reducing total EGR in the reactor. The HI decomposition reactor will be studied using the first two methods to reduce EGR in the reaction system, and the influence of different design parameters on reactor performance will be analyzed. The reference reactor is heated by high-temperature helium gas in a counter-current way, which is closer to the actual industrial reactor. Firstly, the optimal control method will be adopted to solve the optimal reactor performance parameters with the objective of EGRM in the reaction system. Secondly, by changing the inlet temperature and flow rate of helium gas respectively, two other modified reactors with increased length will be designed.

## 2. The System Description

### 2.1. Reactor Model

**Figure 2:**
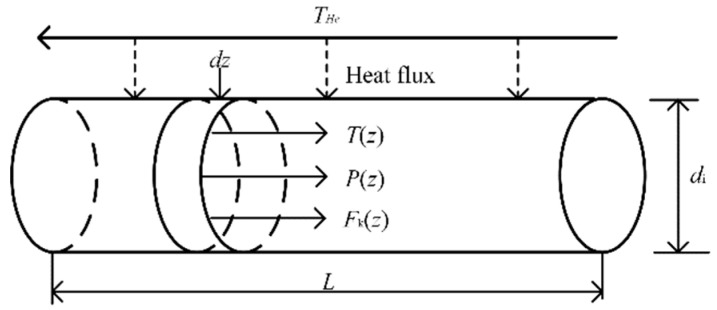
Schematic diagram of the tubular plug flow reactor model.

The chemical reaction equation of HI decomposition reaction is as follows [14,15]:

(1) $$2\mathrm{HI}(g)⇌H_{2}(g){+I}_{2}(g)\;\;\;\;\;\;\;\;\;\Delta H=12\;\mathrm{kJ}/\mathrm{mol}$$

where ΔH is enthalpy of reaction. The reaction is endothermic, and the reaction temperature is usually controlled between 573 and 823 K. The reactor model has an inner diameter of $d_{i}$ and length *L*, the catalyst particles are considered to be ideally spherical and uniformly filled in the tube, and the particle diameter is *d*_p_ The helium temperature, reaction mixture temperature, total pressure, and molar flow rate can be expressed as $T_{He}(z),\;T(z),\;P(z)$ and $F_{k}(z)$, respectively, where z represents the axial position. For the fixed bed reactor with given parameters, the axial back mixing in the reactor can be ignored, and the plug flow model is applicable [15]. Figure 2 shows the schematic diagram of the tubular plug flow reactor model. Meanwhile, the reaction is conducted under high temperature and low pressure operating condition. Therefore, the reaction process can be assumed as follows: (1) The reaction reaches a steady state, no back mixing in the axial direction; (2) The axial and radial mass energy diffusion can be ignored; (3) The reaction mixture gas is considered to be an ideal gas.

The parameters of the reference reactor are listed in Table 1. The inlet conditions of the reactor are determined by the process method for HI decomposition in the SI cycle, and the mixed HI and H_2_O gas are obtained by reaction distillation directly into the decomposition reactor. The inlet composition, inlet temperature, inlet pressure of mixed gas and reactor size listed in Table 1 are all taken from Ref. [15]. Nguyen et al. [15] studied the mechanism of HI decomposition and applied the kinetic model to the reactor design, coupled the helium gas-cooled reactor with the S-I cycle, and the high-temperature helium gas was used as the thermal working medium to heat the HI decomposition reactor. The HI and H_2_O mixture enters the HI decomposition reactor directly from the top of the distiller at a temperature of 468 K and the mixture gas inlet flow rate is 0.6056 mol/s. The high-temperature helium gas flows in the opposite direction at a temperature of 973 K to heat the reaction tube, and the helium inlet flow rate is 3.028 mol/s.

### 2.2. Conservation Equations

The reaction process follows the conservation of energy, mass and momentum. The total heat entering the control unit with an axial direction of dz is used to heat the reaction mixture and to be absorbed by the reaction. The energy conservation equation is [71]:

(2) $$\frac{dT}{dz}=\frac{\pi d_{i}q-\rho_{c}A_{c}(1-\varepsilon )r_{\mathrm{HI}}\Delta H/2}{\sum {}_{k}F_{k}C_{p,k}}$$

where q represents heat flux passing the tube wall, $q=U\left(T_{He}-T\right)$ when the process obeys Newton’s law of cooling, U, which is the overall heat transfer coefficient, can be approximated as a constant value 170 ${W/(m}^{2}\cdot K)$ [15]. $\rho_{c}$ and $A_{c}$ are the catalyst particle density and cross-sectional area of the reaction tube respectively, ε is the void fraction of catalyst bed, and $r_{\mathrm{HI}}$ is the HI decomposition reaction rate, which is strongly dependent on the temperature. $F_{k}$ and $C_{p,k}$ are the molar flow rate and molar specific heat capacity for component k, respectively, and the empirical formula of temperature correlation for $C_{p,k}$ is [94]:

(3) $$C_{p}{{}_{,}}_{k}=A_{k}+B_{k}T+C_{k}T^{2}+D_{k}T^{3}+E_{k}T^{4}$$

where the values of the coefficients $A_{k}{,\;B}_{k}{,\;C}_{k}{,\;D}_{k}$ and $E_{k}$ in the formula are listed in Table 2.

The heat released by the high-temperature helium is used to heat the reactor, so the axial temperature distribution of helium gas along the tube can be expressed as [15]:

(4) $$\frac{dT_{\mathrm{He}}}{dz}=\frac{U\pi d_{i}(T_{\mathrm{He}}-T)}{F_{\mathrm{He}}C_{p,\mathrm{He}}}$$

It means that the helium is heated in a counter-current way, so the temperature of helium gas gradually increases along the positive axis. $C_{p,\mathrm{He}}$ is the molar heat capacity of helium, and the value is 20.786 J/(mol⋅K).

The Reynolds number of the reaction mixture flowing in the fixed bed reactor can be expressed as [80]:

(5) Rep=Gdp/μ

where G is the mass flow rate per unit area of the reaction mixture, $G=\Sigma_{k}(F_{k}M_{k})/A_{c}$, and *μ* is the viscosity of the reaction mixture. To facilitate the calculation, during the reaction *μ* is set as a constant value.

After preliminary calculation, Rep/(1−ε)>500 under the given working parameters, and the pressure drop of the reaction mixture flowing in the tube satisfies the Hick equation [95]:

(6) dPdz=−6.8(1−ε)1.2ε3Rep−0.2ρv2dp

where ρ and v are the density flow velocity of the mixed gas, respectively, $v=F_{T}RT/(PA_{c})$, and R is universal gas constant.

The change of gas composition in the reactor is related to the reaction rate. The HI conversion rate (ξ) and molar flow rate ($F_{k}$) of each component in the mass conservation equation can be expressed as [82]:

(7) $$d\xi /dz=A_{c}\rho_{c}(1-\varepsilon )r_{\mathrm{HI}}/F_{\mathrm{HI},\mathrm{in}}$$

(8) $$dF_{\mathrm{HI}}/dz=-A_{c}\rho_{c}(1-\varepsilon )r_{\mathrm{HI}}$$

(9) $$dF_{H_{{}^{2}}}/dz=A_{c}\rho_{c}(1-\varepsilon )r_{\mathrm{HI}}/2$$

(10) $$dF_{I_{{}^{2}}}/dz=A_{c}\rho_{c}(1-\varepsilon )r_{\mathrm{HI}}/2$$

### 2.3. Chemical Reaction Rate

Shindo et al. [13] deduced the equation for HI decomposition rate based on the assumption that the decomposition of HI on the catalyst surface is a step to determine the reaction rate, and carried out an experimental study on the HI decomposition process with Pt/γ-alumina as catalyst under the conditions of atmospheric pressure. The experimental results showed that the kinetic model can be used to better simulate the reaction process, and the HI decomposition reaction rate equation can be expressed as [13,15]:

(11) $$r_{\mathrm{HI}}=-\frac{dp_{\mathrm{HI}}}{dt}=k\frac{p_{\mathrm{HI}}-\sqrt{p_{I_{2}}p_{H_{2}}}/K_{p}}{{\left[1+\sqrt{K_{I_{2}}p_{H_{2}}}\right]}^{2}}$$

(12) $$k=3.13\times 10^{1}\exp[-E_{a_{1}}/(RT)]$$

(13) $$K_{I_{2}}=1.80\times 10^{-7}\exp[-E_{a_{2}}/(RT)]$$

where k and $K_{I_{2}}$ are the HI decomposition reaction rate constant and iodine adsorption rate constant, respectively, $p_{\mathrm{HI}},p_{H_{2}}$ and $p_{I_{2}}$ are the partial pressures of reaction gas components, and $E_{a_{1}}$ and $E_{a_{2}}$ are the activation energies for the decomposition and adsorption reactions, respectively. When Pt/γ-alumina 1.0 wt% is selected as the catalyst, the values of $E_{a_{1}}$ and $E_{a_{2}}$ are 37.1 × 10^3^ and −75.1 × 10^3^ J/mol [15]. $K_{p}$ and ΔG are the decomposition reaction equilibrium constant and Gibbs free energy changes, and they have the following relationship [96]:

(14) $$K_{p}=\exp(\frac{-\Delta G}{RT})$$

where ΔG is given by JANAF [97].

### 2.4. Model Validation

**Figure 3:**
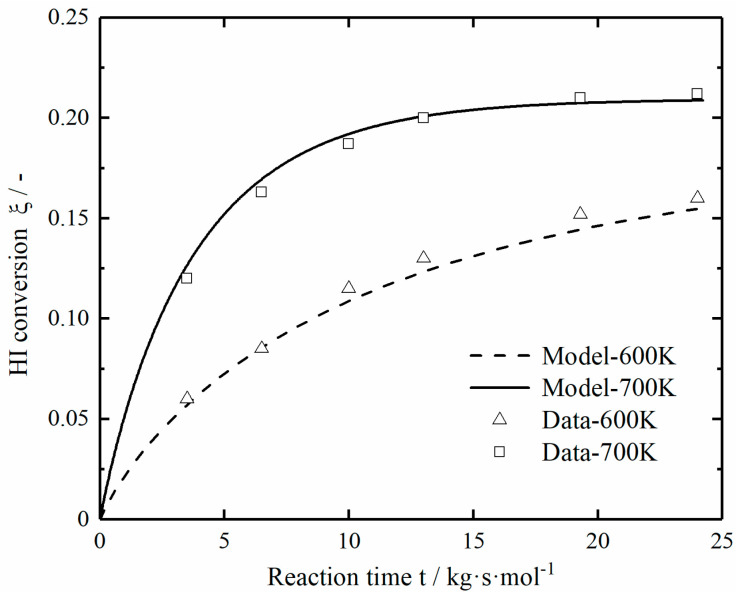
Comparison of numerical simulation values and experimental data values at different temperatures.

Shindo et. al. [13] used a 20 mm tubular fixed-bed reactor for experiments, measured the HI decomposition conversion curves at different temperatures, and the HI solution was passed through the evaporator and entered into the reaction tube for testing during the experiment. Combined with the parameter values in the literature, the numerical calculation results are obtained by applying the established mathematical model of the reactor. Figure 3 shows the comparison between the numerical calculation results and the experimental data values at different temperatures. When the decomposition temperature is 600 and 700 K, the simulation results are in good agreement with the experimental data, and the maximum relative percentage error is 5.5%. This indicates that the experimental results can be accurately predicted by the established reactor model.

### 2.5. Entropy Generation Rate

According to the theory of non-equilibrium thermodynamics, the local EGRs in the reactor are produced by three processes of heat transfer, chemical reaction and fluid flow [98,99], and the local total EGR can be expressed as:

(15) $$\begin{array}{ll} \sigma_{\mathrm{tot}}= & \sigma_{\mathrm{ht}}+\sigma_{\mathrm{cr}}+\sigma_{f}\\ = & \pi d_{i}q(\frac{1}{T}-\frac{1}{T_{\mathrm{He}}})+A_{c}\rho_{c}(1-\varepsilon )r(-\frac{\Delta_{r}G}{T})+A_{c}v\left[-\frac{1}{T}\left(\frac{dP}{dz}\right)\right] \end{array}$$

where $\sigma_{\mathrm{ht}}$, $\sigma_{\mathrm{cr}}$ and $\sigma_{f}$ represent the local EGRs caused by heat transfer, chemical reaction and fluid flow, respectively. Each term is the product of flux and force of the corresponding transport process.

The thermodynamic driving force of the heat transfer process is:

(16) $$\Delta \left(\frac{1}{T}\right)=\frac{1}{T}-\frac{1}{T_{\mathrm{He}}}$$

The chemical driving force of the chemical reaction process is:

(17) $$-\frac{\Delta_{r}G}{T}=-R\ln\frac{p_{H_{2}}{}^{0.5}p_{I_{2}}{}^{0.5}}{p_{\mathrm{HI}}K_{p}}$$

The viscous driving force of fluid flow process is:

(18) $$-\frac{1}{T}\left(\frac{dP}{dz}\right)$$

The total EGR is the local EGR integral in the length of the reaction tube:

(19) $${\left(dS/dt\right)}_{\mathrm{tot}}=\int_{0}^{L}\sigma_{\mathrm{tot}}dz$$

In addition, according to the entropy balance equation, the total EGR of the reaction system can also be calculated using the following formula [77]:

(20) $${\left(dS/dt\right)}_{\mathrm{tot}}=F_{T,\mathrm{out}}S_{\mathrm{out}}-F_{T,\mathrm{in}}S_{in}-\pi d_{i}\int_{0}^{L}\frac{q(z)}{T_{He}}dz$$

where $S_{\mathrm{in}}$ and $S_{\mathrm{out}}$ represent the molar entropy flow at the reactor inlet and outlet respectively, and the last term is the heat entropy flow of high temperature helium gas to the reaction system. Equation (20) can be used to verify the correctness of Equation (19) in calculating the total EGR, and shows that heating with lower temperature helium can help reduce the total EGR in the reactor under the same import and export conditions.

## 3. The Optimization Problem

In the chemical production process, the reaction product is usually required to meet a certain output, so the $H_{2}$ PR is kept constant during the optimization process. The optimization problem can be described as solving the optimal $T_{\mathrm{He}}(z)$ to minimize the total EGR in the HI decomposition reactor under the constraint of fixed $H_{2}$ PR. The constraints of the optimization problem can be divided into two categories: one is the energy, momentum and mass balance Equations (2), (6) and (7), which restrict the change of state variables, and another is the inlet and outlet boundary conditions, such as temperature, pressure, and conversion rate at the reactor inlet and outlet.

### 3.1. The Optimal Control Formulation

According to optimal control theory, the Hamilton function H of the optimal control problem is [77,78,100]:

(21) $$H[x(z),\lambda (z),u(z)]=\sigma_{\mathrm{tot}}[x(z),u(z)]+\sum_{i=1}^{3}\lambda_{i}(z)f_{i}\left[x(z),u(z)\right]$$

where x=[T(z),P(z),ξ(z)] is the state variable vector, $\lambda =[\lambda_{T},\lambda_{P},\lambda_{\xi }]$ is the covariate variable vector, and $f_{i}$ represents the corresponding conservation equation (Equations (2), (6) and (7)), the control variable is the external helium temperature $T_{\mathrm{He}}(z)$ i.e., $u(z)=T_{\mathrm{He}}(z)$.

Based on the Pontryagin minimum value principle [101], the following necessary conditions should be met when the objective function takes the minimum value:

(22) $$dT/dz=\partial H/\partial \lambda_{T}$$

(23) $$dP/dz=\partial H/\partial \lambda_{P}$$

(24) dξ/dz=∂H/∂ξ

(25) $$d\lambda_{T}/dz=-\partial H/\partial T$$

(26) $$d\lambda_{P}/dz=-\partial H/\partial P$$

(27) $$d\lambda_{\xi }/dz=-\partial H/\partial \xi$$

When the Hamiltonian function H takes the extreme value, the control variable satisfies the following equation:

(28) $$dH/dT_{\mathrm{He}}=0$$

In the Hamiltonian function, H, only the local EGR of heat transfer in Equations (15) and the energy conservation equation in Equation (2) are related to $T_{\mathrm{He}}$. By omitting other terms not related to $T_{\mathrm{He}}$, the Hamiltonian function can be written as:

(29) $$H\left(T_{\mathrm{He}}\right)=\pi d_{i}q(\frac{1}{T}-\frac{1}{T_{\mathrm{He}}})+\lambda_{T}\frac{\pi d_{i}q}{\Sigma_{k}F_{k}C_{p,k}}$$

Substituting $q=U(T_{\mathrm{He}}-T)$ into Equations (29) and continuing to omit other items irrelevant to $T_{\mathrm{He}}$ yields:

(30) $$H\left(T_{\mathrm{He}}\right)=\frac{T_{\mathrm{He}}}{T}-\frac{T}{T_{\mathrm{He}}}+\frac{\lambda_{T}T_{\mathrm{He}}}{\Sigma_{k}F_{k}C_{p,k}}$$

The optimal helium temperature $T_{\mathrm{He}}(z)$ can be obtained by solving Equations (28) and (30):

(31) $$T_{\mathrm{He}}=T{\left(1+\frac{\lambda_{T}T}{\Sigma_{k}F_{k}C_{p,k}}\right)}^{-1/2}$$

### 3.2. Boundary Conditions

The optimal control boundary conditions can be specified or are left free. When the state variable is free, the corresponding coordinator variable is 0. According to the second type of boundary condition constraints mentioned earlier, optimal reactor has the same hydrogen production rate, inlet temperature, inlet and outlet pressure as the reference reactor, so the boundary conditions can be summarized as:

(32) $$\xi (0)=0\;\mathrm{and}\;\xi (L)=\xi_{L}^{\mathrm{ref}}$$

(33) $$T(0)\;=T_{0}^{\mathrm{ref}}\;\mathrm{and}\;\lambda_{T}(L)=0$$

(34) $$P(0)=P_{0}^{\mathrm{ref}}\;\mathrm{and}\;P(L)=P_{L}^{\mathrm{ref}}$$

In the above boundary condition, $T_{0}^{\mathrm{ref}}$ and $P_{0}^{\mathrm{ref}}$ are 468 K and 7 bar, respectively. $\xi_{L}^{\mathrm{ref}}$ and $P_{L}^{\mathrm{ref}}$ are obtained by solving the reference reactor.

### 3.3. Numerical Solution Metho

The optimal control problem includes 6 differential equations (Equations (22)–(27)), 1 algebraic equation (Equation (31)), and 6 boundary conditions (Equations (32)–(34)), which can be turned into the boundary value problem of the differential equations, and solved by Matlab solver ‘bvp4c’. The initial value has a great influence on the calculation result when ‘bvp4c’ is used to solve the optimal problem, the nonlinear programming method proposed by Nummedal et. al. [74] is used to discretize various constraint equations and the objective function, and the Matlab solver ‘fmincon’ is used to select the initial 100 grids for calculation to obtain the initial value of discrete points. To meet the calculation accuracy, 3000 grids are selected to solve the function of ‘bvp4c’, the calculation accuracy at this point is greater than 2 × 10^−10^.

## 4. Numerical Results and Discussions

### 4.1. The Reference Reactor

**Figure 4:**
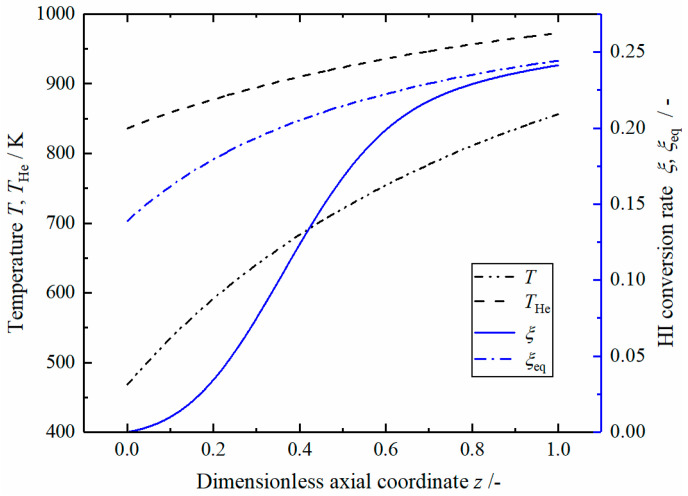
The temperature and hydrogen iodide (HI) conversion rate in the reference reactor.

Figure 4 shows the profiles of reaction mixture temperature T and helium temperature $T_{\mathrm{He}}$, the actual HI conversion rate and HI equilibrium conversion rate in the reference reactor. High temperature helium flows in the reverse direction, and its temperature drops almost linearly from 973 to 836 K at the outlet. The reaction mixture temperature increases rapidly at the inlet, then the growth rate gradually slows down, and increases to 856 K at the outlet. The pressure dropped by 0.135 bar. The HI decomposition conversion reaches 0.241 at the outlet; it increases rapidly in the middle position (0.2<z<0.6), this indicates a relatively high reaction rate. The difference between the equilibrium conversion rate and the actual conversion rate gradually decreases and remains constant at the end of the reaction tube, and this phenomenon indicates that the reaction driving force keeps constant while approaching the equilibrium state.

**Figure 5:**
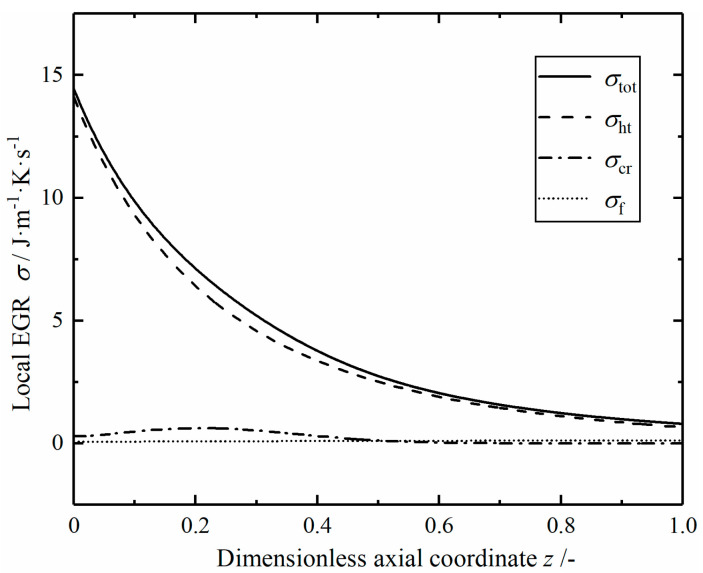
The local energy generation rates (EGRs) in the reference reactor.

Figure 5 shows the profiles of local EGRs in the reference reactor. The local total EGR $\sigma_{\mathrm{tot}}$ consists of three parts. The local EGR of heat transfer is relatively large, especially at the inlet, followed by the chemical reaction component, and the fluid flow component is relatively small. The local total EGR gradually decreases with the increase of dimensionless length, which is consistent with the change trend of heat transfer component. This is because with the increase of tube length, the temperature difference between helium and mixed gas gradually decreases, resulting in the decrease of the heat transfer driving force. The local EGR $\sigma_{\mathrm{cr}}$ of chemical reaction increases first and then decreases gradually from the reactor inlet. This is because the low temperature at the inlet leads to a small reaction rate, and there is a small chemical driving force near the reaction equilibrium state at the outlet, so the interaction of flux and force in the chemical reaction makes $\sigma_{\mathrm{cr}}$ reach the maximum value in the middle part. The local EGR $\sigma_{f}$ of fluid flow increases slowly; this is due to the increase of reaction temperature and the acceleration of gas velocity, which leads to the increase of pressure drop rate.

### 4.2. The Optimal Reactor

**Figure 6:**
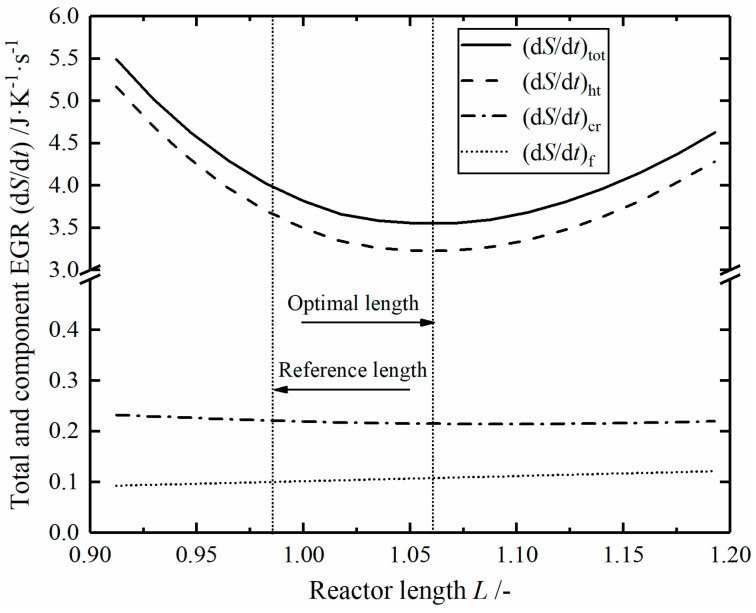
Relationships of the total and component EGRs versus the reactor length *L.*

The optimal and reference reactors have the same pressure drop, and the relationships of total and three components EGRs versus the length of the reaction tube are shown in Figure 6. The total EGR of heat transfer process decreases first and then increases, while the total EGRs due to chemical reaction and fluid flow are almost constants. When the minimum total EGR is obtained, the corresponding length of the reaction tube is 1.059 m.

**Figure 7:**
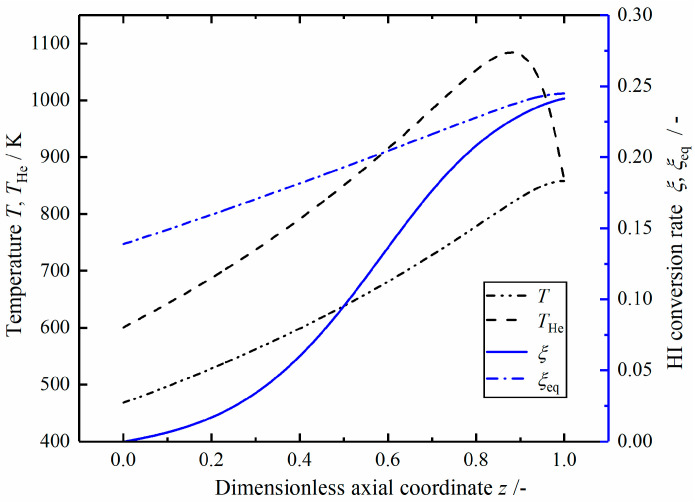
The temperature and HI conversation rate in the optimal reactor.

Figure 7 shows the profiles of temperature and HI conversion rate in the optimal reactor. The helium gas temperature increases from 600 to 1084 K, and then quickly drops to 858 K at the outlet. The temperature of the mixed gas increases gradually from the inlet. The difference between temperatures of helium and the mixed gas increases slowly at first, and then gradually decreases to 0 at the outlet. When the helium temperature is near the maximum, the two temperatures have the maximum difference, and the end of the temperature curve is closed due to the free temperature boundary conditions at the outlet. The difference between the equilibrium conversion rate and the actual conversion rate decreases monotonically in the axial direction, the actual conversion rate is close to the equilibrium conversion rate at the outlet.

**Figure 8:**
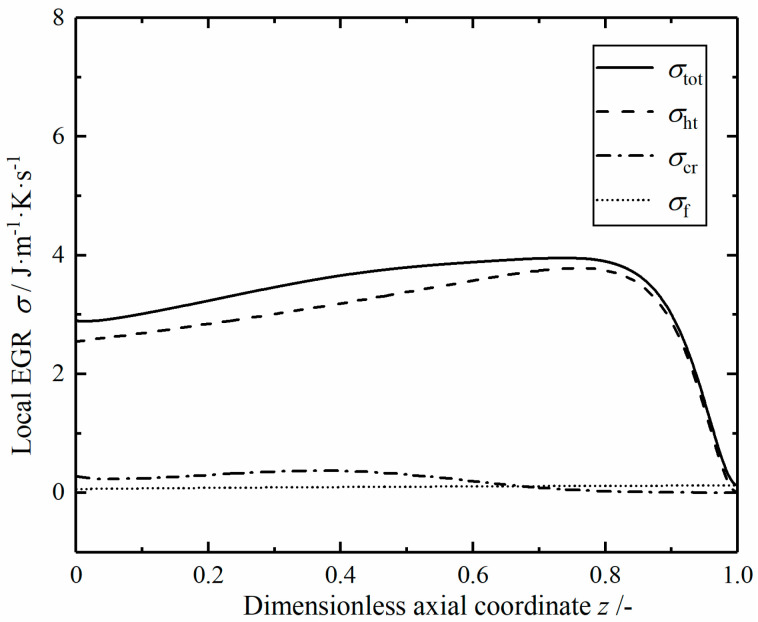
The local EGRs in the optimal reactor.

Figure 8 shows the local EGRs profiles in the optimal reactor. The local EGR contributed by the heat transfer process plays a dominant role; it keeps a certain value at the inlet and remains relatively stable, then drops sharply at the outlet. The local EGR due to the chemical reaction remains stable in the front of the reactor and shows a relatively uniform distribution. The local EGR due to fluid flow increases slowly with increasing temperature. Local total and heat transfer EGRs have similar trends; they are evenly distributed in the length of reaction tube except at the inlet and outlet.

### 4.3. Modified Reactors

The design requirements of the modified reactor are as follows: under the constraint of fixed hydrogen PR, the length of the reactor and the inlet conditions of the helium gas are changed to achieve the same total EGR as that of the optimal reactor. The two types of modified reactors designed are realized by changing the inlet temperature and inlet flow rate of helium gas respectively, which are denoted as “Case 1” and “Case 2” reactors; the remaining parameter values in the modified reactor remain the same as reference values. In order to clearly compare the differences between the reactor design parameters, the design parameters of the reference reactor, optimal reactor and modified reactors are listed in Table 3. In the table, the “reference value” (RV) indicates that the parameter is consistent with that of the reference reactor, and the “optimal value” (OV) and the “calculated value” (CV) indicate that the parameters are obtained by optimization and calculation methods, respectively.

#### 4.3.1. Case 1 Reactor

**Figure 9:**
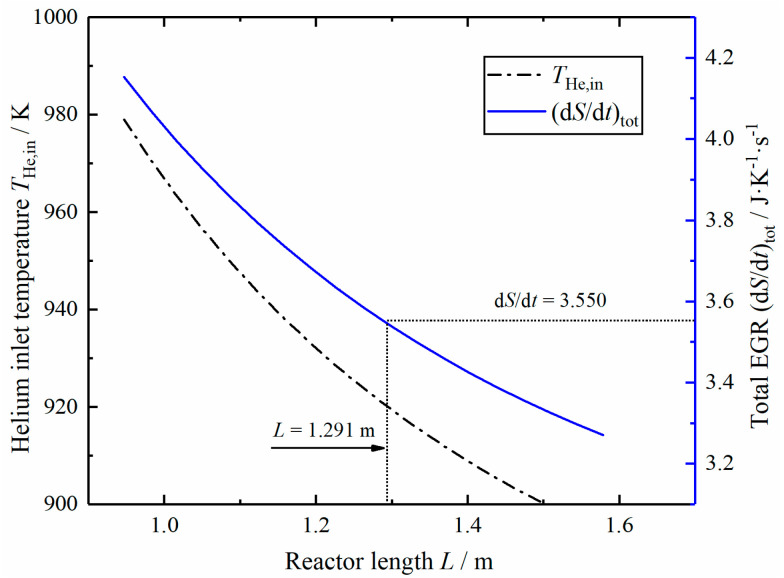
Relationships of the helium inlet temperature and total EGR versus the reactor length L.

Figure 9 shows the curve of inlet helium temperature and total EGR changing with reactor length L under the same hydrogen production rate. As the length increases, the corresponding helium inlet temperature decreases, and the total EGR also decreases gradually. In order to achieve the optimal value of total EGR as that in optimal reactor, the Case 1 reactor length should be extended to 1.291 m and the corresponding helium inlet temperature drops from 973 to 919 K.

**Figure 10:**
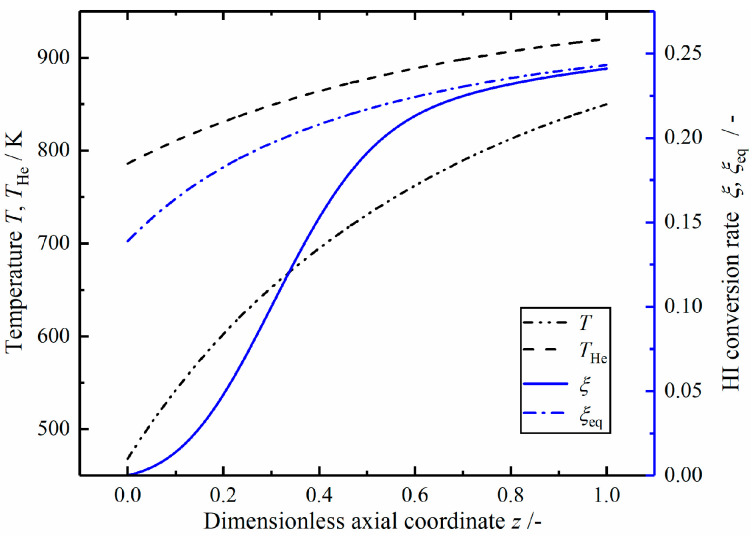
The temperature and HI conversion rate in the Case 1 reactor.

Figure 10 shows the variation curves of temperature and HI conversion rate in the Case 1 modified reactor. The outlet temperatures of helium and reaction mixture are 784 and 850 K, respectively. The actual conversion rate increases rapidly in the front part, approaches close to the equilibrium conversion rate at z=0.6, and then begins to increase slowly along the equilibrium curve.

**Figure 11:**
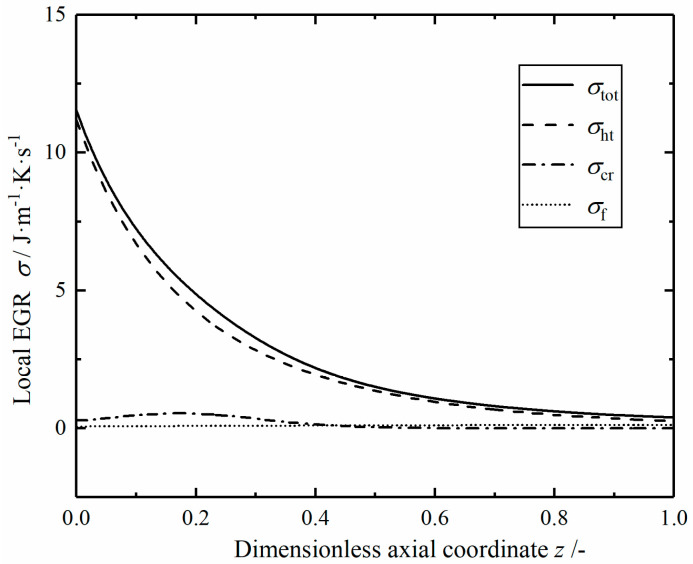
The local EGRs in the Case 1 reactor.

Figure 11 shows the profiles of local EGRs in the Case 1 modified reactor. The local EGR contributed by the heat transfer process still accounts for a large proportion, and gradually decreases in the axial direction. The local EGR contributed by the chemical reaction first increases to the maximum value and then decreases gradually while the local EGR caused by fluid flow increases slowly. The overall change of local EGRs is similar to that of the reference reactor.

#### 4.3.2. Case 2 Reactor

**Figure 12:**
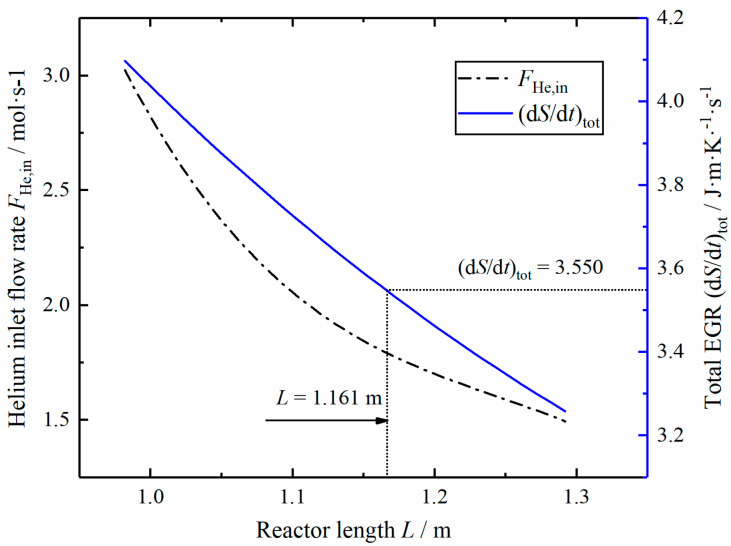
Relationships of the helium inlet flow rate and total EGR versus the reactor length *L.*

Figure 12 shows the curves of inlet helium flow rate and total EGR changing with reactor length *L* under the same hydrogen PR. The corresponding helium flow rate and total EGR decrease when the length increases. When the total EGR of Case 2 reactor is the same as the optimal value, the length L of the reactor extends to 1.161 m, and the helium inlet flow rate is 1.788 mol/s.

**Figure 13:**
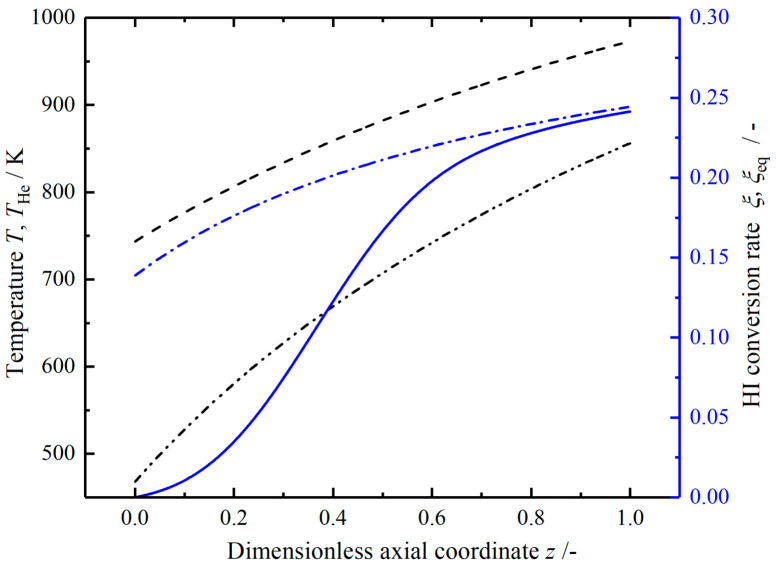
The temperature and HI conversion rate in the Case 2 reactor.

Figure 13 shows the variation curves of temperature and HI conversion rate in the Case 2 modified reactor. The helium temperature gradually dropped from 973 to 743 K, the mixture temperature at the outlet is 856 K, and the HI actual conversion rate increases slowly after approaching the equilibrium conversion rate at the dimensionless position z=0.7.

**Figure 14:**
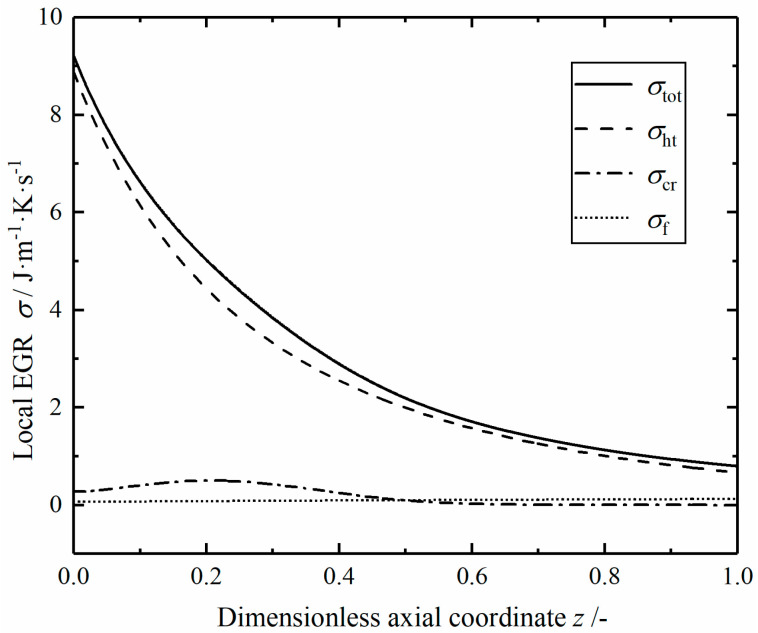
The local EGRs in the Case 2 reactor.

Figure 14 shows the profiles of local EGRs in the Case 2 modified reactor. The variations of local EGRs are similar to those of the Case 1 and reference reactor, but with slightly different values. The local EGR of heat transfer decreases monotonically, and local EGR of fluid flow increases slowly. The local EGR of chemical reaction reaches its maximum value at z=0.2.

### 4.4. Discussions

Table 4 lists the total EGR and component EGR values in the reactors. The total EGR of both optimal and modified reactors decreased by 13.3% compared with the reference value; the decrease of total EGR is mainly caused by the decrease of EGR in the heat transfer process. Compared with the reference value, the total EGR contributed by the heat transfer process in the three reactors decreased by 14.4%, 15.1% and 14.7%, respectively, and the total EGRs of fluid flow component in two modified reactors increase obviously, which is mainly due to the large pressure drop brought by the increase of tube length. Therefore, the reduction of total EGR in the reaction system can be achieved by changing the temperature distribution of the thermal fluid or by extending the length of the reactor; both methods are achieved by reducing the EGR in the heat transfer process, but extending the reactor length usually results in a large pressure loss.

**Figure 15:**
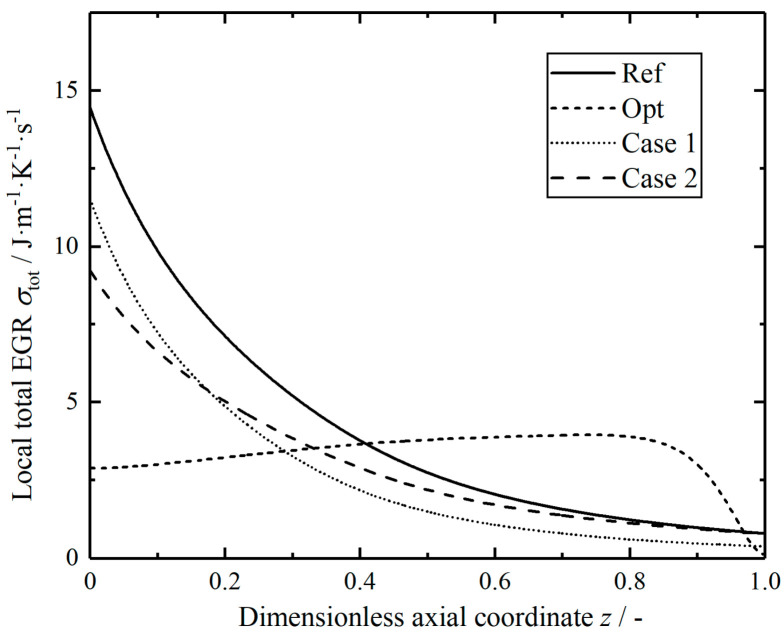
Comparison of the local total EGRs in the reactors.

Figure 15 shows the distributions of the local total EGRs in the reactors along the dimensionless axial position. It is obvious that the local total EGR of the optimal reactor is most evenly distributed in the axial position, this distribution can be analyzed and understood by the equipartition of entropy production theory proposed by Johannessen and Kjelstrup [77]; the local EGR tends to be a constant in the middle part in the optimal control system, it changes dramatically at the outlet due to the outlet fixed boundary conditions. The variation trends of local total EGRs in two modified reactors are similar to that in the reference reactor, however, the change of curve is relatively flat, and the corresponding value of the axial position is also relatively small.

Vander Ham et al. [100] proposed to use the thermodynamic performance indicator and equipartition indicator to evaluate the EGR in chemical reactors, where the equipartition indicator is measured by the change of the local EGR. The standard deviation $\sigma_{\mathrm{tot}}$ is calculated as follows [100]:

(35) $$s=\sqrt{\frac{1}{n}\sum_{i=1}^{n}{\left(\sigma_{z,i}-m\right)}^{2}}$$

where the discrete n data points were used to solve the standard deviation s of the local EGR curve, and m is the average of these data points. The dimensionless coefficient of variation c is the ratio of the standard deviation *s* to the average *m*, i.e., c=s/m, which is used to measure the degree of equalization, so the closer the curve distribution is to the equipartition state, the smaller is the c value. Table 5 lists the comparison of equipartition indicator in the reactors. It can be seen that the optimal reactor has the best equalization characteristics, and the Case 1 reactor is relatively poor. As the EGR of heat transfer accounts for a large proportion, it is an important factor affecting the equipartition indicator of local total EGR.

**Figure 16:**
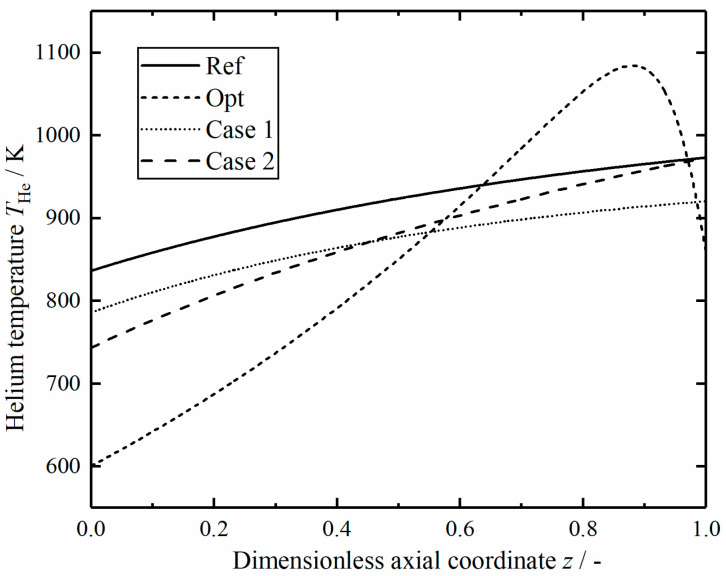
Comparison of the helium gas temperature in the reactors.

Figure 16 shows the comparison of helium temperature in the reactors along dimensionless axial position. Except for the optimal reactor, the helium temperature in the other three reactors varies monotonously along the axial direction. The helium temperature in the optimal reactor increases gradually from a low temperature value to a maximum value and then drops sharply; the helium flow rate required to achieve the temperature profile is freely variable in the axial direction. Therefore, the results indicate that it is advantageous to use a lower temperature in the reactor inlet to reduce the entropy generation rate.

**Figure 17:**
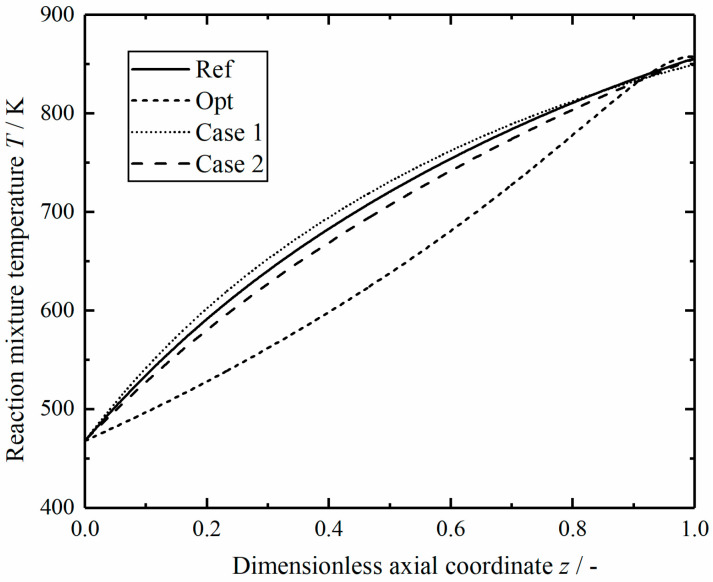
Comparison of the reaction mixture temperature in the reactors.

Figure 17 shows the comparison of reaction mixture temperature in the reactors along dimensionless axial position. The temperature of reaction mixture in the optimal reactor increases slowly at the beginning and gradually surpass temperature curve in other reactors at z=0.9. The temperature variations of reaction mixture in the modified reactors and the reference reactor are similar.

**Figure 18:**
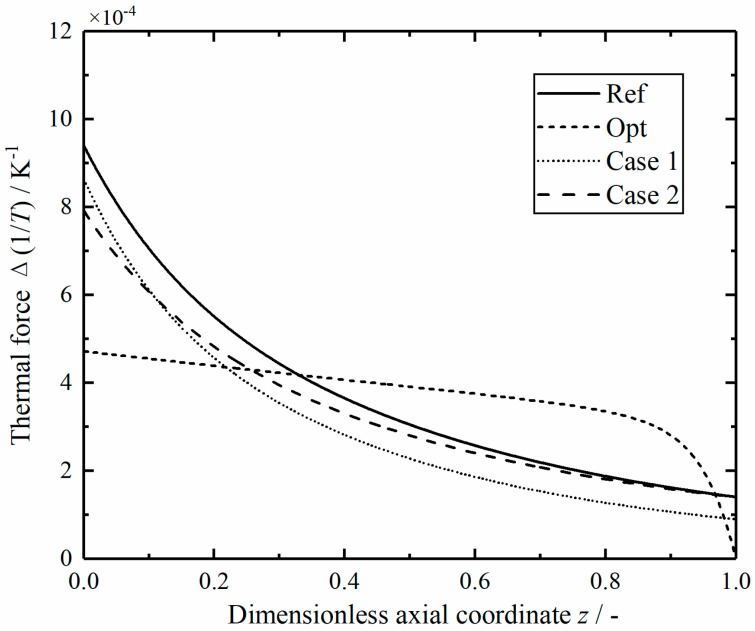
Comparison of the thermal driving forces in the reactors.

Figure 18 shows the comparison of thermal driving forces in the reactors along the dimensionless axial position. All the thermal driving forces gradually decrease from the maximum value at the inlet, but the thermal driving forces of the optimal reactor are relatively flat at the middle position, and decrease sharply at the outlet. This is because there is a relatively constant temperature difference between the helium and the mixed gas in the optimal reactor, as shown in Figure 7.

**Figure 19:**
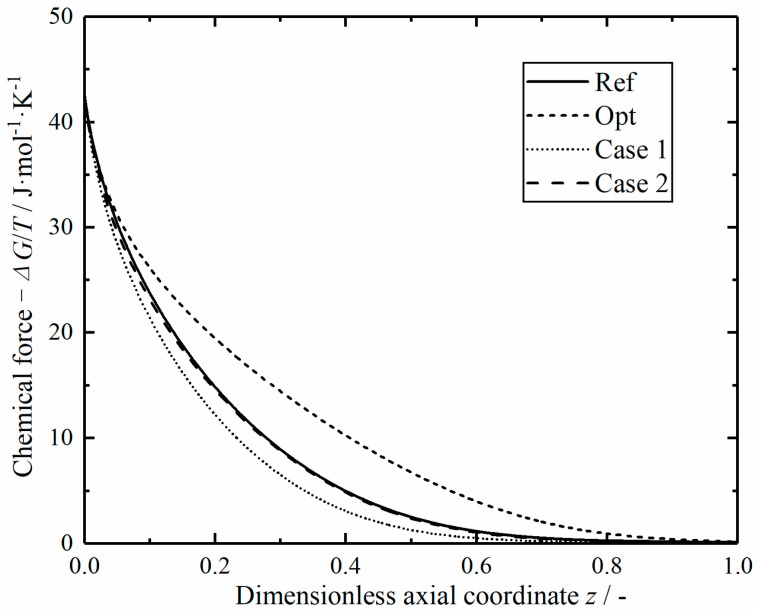
Comparison of the chemical driving force in the reactors.

Figure 19 shows the comparison of chemical driving forces in the reactors along dimensionless axial position. All the chemical reaction driving forces decrease sharply at the inlet, and the chemical driving force of the optimal reactor is relatively high in the middle section. This is because the mixture temperature in the inlet section of the optimal reactor is lower, and the reaction is far away from the equilibrium state, so a large chemical driving force is generated.

**Figure 20:**
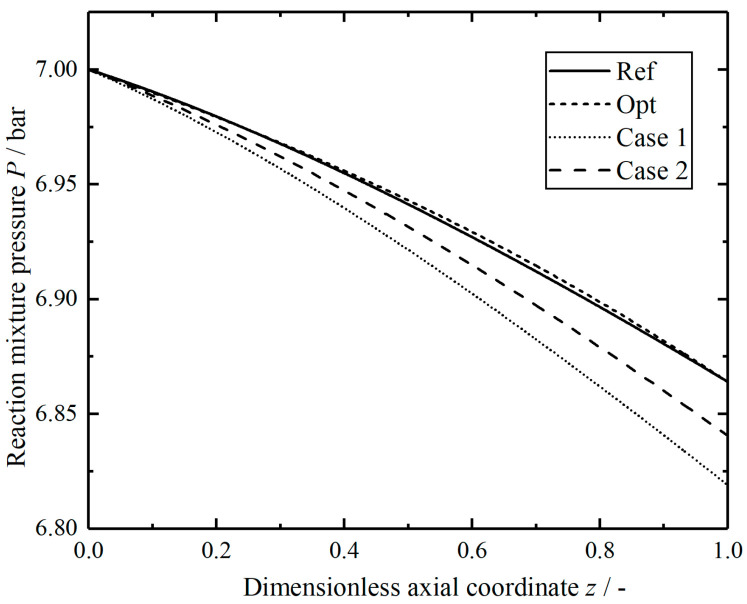
Comparison of the pressure in the reactors.

Figure 20 shows the comparison of the pressures in the reactors along dimensionless axial position. Although the optimal reactor length increases slightly compared with the reference length, the pressure drop in the tube remains the same. Compared with the reference values, the lengths of two modified reactors increase by 31.5% and 18.6%, respectively, and the pressure drops increase by 33.1% and 17.6%, respectively. When the length of the modified reactor increases appropriately, helium gas with lower temperature and lower flow rate can be selected for heating to reduce the total EGR in decomposition reaction, but the increase of reaction tube length will cause greater pressure loss, which requires a more powerful compressor to supercharge.

## 5. Conclusions

A one-dimensional tubular plug flow reactor model for HI decomposition is established based on FTT theory, and the reference reactor is heated by helium countercurrent. Based on the reference reactor design parameters, the optimal reactor and the modified reactors with different parameters are designed by adjusting the distribution of EGR and extending the length of the reactor, and the total EGR of those reactors are reduced by 13.3% compared with the reference value; the reduction of total EGR is mainly caused by the decrease of EGR in heat transfer process. The optimal reactor is solved by the optimal control theory, and the distribution of local total EGR is more uniform, the optimal helium temperature profile is ideal and requires a lower temperature at the reactor inlet. The length of the modified reactors increases significantly compared with the reference reactor length, and the local EGR distribution remains similar to that of the reference reactor. However, the increase of length will bring an additional pressure drop, which requires a more powerful compressor to compensate for the pressure. The method of optimizing the temperature profile of helium can reduce the total EGR without increasing the pressure drop in the reactor, but achieving this temperature profile in actual production practice requires improvement of corresponding heating equipment. The results obtained herein can provide some guidelines for the design of HI decomposition reactor structure parameters.

## Figures and Tables

**Table 1:** The parameters of the reference reactor [15].

Parameters	Symbol	Value
Inlet temperature of reaction mixture	$T_{0}$	468 K
Helium gas inlet temperature	$T_{\mathrm{He},\mathrm{in}}$	973 K
Overall heat transfer coefficient	U	$170\;W/(K\cdot m^{2})$
Mixed gas viscosity	μ	$3.4\times 10^{-5}\;\mathrm{kg}/(m\cdot s)$
Inlet pressure	$P_{0}$	7 bar
Catalyst particle density	$\rho_{c}$	$1250{\;\mathrm{kg}/m}^{3}$
Porosity of catalyst bed	ε	0.5
Catalyst particle diameter	$d_{p}$	$3\times 10^{-3}\;\mathrm{mol}/s$
Helium gas inlet flow rate	$F_{\mathrm{He},\mathrm{in}}$	3.028 mol/s
Inlet total flow rate	$F_{T,\mathrm{in}}$	0.6056 mol/s
Inlet HI molar fraction	$x_{\mathrm{HI},\mathrm{in}}$	20.48%
Inlet H_2_ molar fraction	$x_{H_{2},\mathrm{in}}$	0.01%
Inlet I_2_ molar fraction	$x_{I_{2},\mathrm{in}}$	0.01%
Inlet H_2_O molar fraction	$x_{H_{2}O,\mathrm{in}}$	79.5%
Reactor inner diameter	$d_{i}$	0.0762 m
Reactor length	L	0.982 m

**Table 2:** Thermodynamic parameter values of components [94].

Parameters	k=HI	$k=H_{2}O$	$k=H_{2}$	$k=I_{2}$
$A_{k}$	29.770	33.933	25.399	34.151
$B_{k}(\times 10^{-3})$	−7.4945	−8.4186	20.178	13.930
$C_{k}(\times 10^{-5})$	2.0687	−2.9906	−3.8549	−2.0952
$D_{k}(\times 10^{-8})$	−1.1963	−1.7825	3.1880	1.4362
$E_{k}(\times 10^{-12})$	2.1010	3.6934	−8.7585	−3.5948
$M_{k}\times 10^{3}\;(\mathrm{kg}/\mathrm{mol})$	127.912	18.015	2.016	253.809

**Table 3:** Comparison of reactor design parameters.

Parameter	Ref	Opt	Case 1	Case 2
$T_{\mathrm{He},\mathrm{in}}$	RV	OV	CV	RV
$F_{\mathrm{He},\mathrm{in}}$	RV	-	RV	CV
ξ(L)	RV	RV	RV	RV
P(L)	RV	RV	CV	CV
T(L)	RV	OV	CV	CV
L	RV	OV	CV	CV
${(dS/dt)}_{\mathrm{tot}}$	RV	OV	fixed at OV	fixed at OV

**Table 4:** Comparison of the total and component EGRs in the reactors (J·K^−1^·s^−1^).

Parameter	Ref	Opt	Case 1	Case 2
L (m)	0.982	1.059	1.291	1.165
Heat transfer	3.772	3.228	3.201	3.218
Chemical reaction	0.221	0.216	0.217	0.216
Fluid flow	0.099	0.108	0.132	0.116
Total	4.092	3.550	3.550	3.550

**Table 5:** Comparison of equipartition indicator in the reactors.

Indicator	Ref	Opt	Case 1	Case 2
$c_{\mathrm{tot}}$	0.8462	0.2167	1.0263	0.7269
$c_{\mathrm{ht}}$	0.8715	0.2420	1.0751	0.7492
$c_{\mathrm{cr}}$	1.1050	0.7245	1.3079	1.1172
$c_{f}$	0.1678	0.1959	0.1669	0.1732

## Nomenclature

$A_{c}$	Cross-section area: $m^{2}$
$C_{p}$	Molar heat capacity, $J\cdot {\mathrm{mol}}^{-1}\cdot K^{-1}$
c	Dimensionless coefficient of variation
d	Diameter, m
G	Mass flow rate per unit area, ${\mathrm{kg}\;s}^{-1}m^{-2}$
F	Molar flow rate, $\mathrm{mol}\cdot s^{-1}$
H	Hamiltonian function of the optimal problem
$K_{I_{2}}$	Adsorption equilibrium constant of iodine, ${\mathrm{bar}}^{-1}$
$K_{p}$	Equilibrium constant
k	Reaction rate constant, ${\mathrm{mol}\;s}^{-1}{\mathrm{kg}}^{-1}$
L	Length, m
M	Molar mass, ${\mathrm{kg}\;\mathrm{mol}}^{-1}$
P	Total pressure, bar
p	Partial pressure of reaction component gas, bar
q	Heat flow per unit area, ${W\;m}^{-2}$
U	Overall heat transfer coefficient, $W\;m^{-2}{\;K}^{-1}$
u	Control variable
v	Superficial velocity, ${m\;s}^{-1}$
R	Universal gas constant, ${J\;\mathrm{mol}\;K}^{-1}$
Re	Reynolds number
r	Chemical reaction rate, ${\mathrm{mol}\;s}^{-1}{\;\mathrm{kg}}^{-1}$
S	Entropy, ${J\;K}^{-1}{\;\mathrm{mol}}^{-1}$
(dS/dt)	Entropy generation rate, ${J\;K}^{-1}{\;s}^{-1}$
T	Temperature, K
x	Gas mole fraction
x	State variable vector
z	Axial coordinate

## Greek Symbol

ε	Porosity
μ	Dynamic viscosity, ${\mathrm{kg}\;m}^{-1}{\;s}^{-1}$
σ	Local entropy generate rate, ${J\;K}^{-1}{\;m}^{-1}{\;s}^{-1}$
ξ	Conversion rate
ρ	Density, $\mathrm{kg}\cdot m^{-3}$
λ	Covariate variable vector
ΔG	Gibbs free energy change of reaction, ${J\;\mathrm{mol}}^{-1}$
ΔH	Enthalpy of reaction, ${J\;\mathrm{mol}}^{-1}$

## Subscripts

c	Catalyst particle
i	Internal of reactor
k	Component k
in	Inlet
out	Outlet
p	Particle
0, L	Axial position of the reactor

## Superscripts

ref	Reference reactor

## Abbreviations

CV	Calculated value
EGM	Entropy generation minimization
EGR	Entropy generation rate
EGRM	Entropy generation rate minimization
FTT	Finite-time thermodynamics
HI	Hydrogen iodide
OV	Optimal value
PR	Production rate
PRM	Production rate maximization
RV	Reference value
S-I	Sulfur-iodine
